# Molecularly Imprinted Polymers-Coated CdTe Quantum Dots for Highly Sensitive and Selective Fluorescent Determination of Ferulic Acid

**DOI:** 10.1155/2019/1505878

**Published:** 2019-07-08

**Authors:** Zhihong Wang, Ruiqing Long, Mijun Peng, Te Li, Shuyun Shi

**Affiliations:** ^1^Guangdong Provincial Public Laboratory of Analysis and Testing Technology, Guangdong Institute of Analysis, Guangzhou 510070, China; ^2^College of Chemistry and Chemical Engineering, Central South University, Changsha 410083, China; ^3^Key Laboratory of Hunan Province for Water Environment and Agriculture Product Safety, Central South University, Changsha 410083, China

## Abstract

Ferulic acid (FA), an important phenolic acid, is widely distributed in higher plants and presents many pharmacological effects. Therefore, sensitive determination of FA in complex matrix is necessary. Molecularly imprinted polymers-coated CdTe quantum dots (CdTe-QDs@MIPs) exhibited incomparable advantages because of their combination of excellent selectivity of MIPs and high sensitivity of QDs. Here, a fluorescent probe based on CdTe-QDs@MIPs was successfully fabricated for selective and sensitive determination of FA. MIPs shell was obtained by the reverse microemulsion method using FA, 3-(aminopropyl) triethoxysilane (APTES), and tetraethyl orthosilicate (TEOS), as template, functional monomer, and crosslinker. In optimal conditions, the fluorescence CdTe-QDs@MIPs sensor exhibited fast response (within only 3 min), high sensitivity (limit of detection, LOD at 0.85 *μ*g/l), excellent linear ranges (2–100 *μ*g/l) with a correlation coefficient of 0.9996, and distinguished selectivity for FA. Satisfactory recoveries from 91.8% to 110.3% were achieved with precisions below 6.6% for FA analysis in real pineapple juice and apple juice by developed CdTe-QDs@MIPs. The fluorescence results coincided well with those obtained by high-performance liquid chromatography (HPLC). It could be concluded that the resultant CdTe-QDs@MIPs offered a new way for rapid and sensitive analysis of FA in the complex matrix.

## 1. Introduction

Ferulic acid (FA), one of the best-known natural phenolic acids, widely distributes in grains, vegetables, fruits, alcoholic beverages, and herbs (e.g., rice, wheat, barley, onion, orange, tomato, corn, pineapple, beer, *Ferula asafoetida*, *Ligusticum chuanxiong*, *Lycopodium selago*, and *Equisetum hiemale*) [1]. FA exhibits numerous biological activities, such as antioxidant, anticancer, antimicrobial, anti-inflammatory, and antidiabetic effects [1, 2]. Moreover, FA has been proved effective in protecting skin from the onset of erythema from UVB rays [3]. It is therefore necessary to quantify FA [4]. For the sensitive determination of FA, several methods have been developed including high-performance liquid chromatography (HPLC) [5, 6], electrochemical techniques [7, 8], Raman spectroscopy [9], chemiluminescence [10], and fluorescence [11]. However, some of these methods need professional operators and/or sophisticated instruments.

Quantum dots (QDs) exhibited unique optical and chemical features, such as facile preparation procedures, size- and composition-tunable photoluminescence-emission modifiable surfaces, high quantum yield, good chemical and photo stability, and superior aqueous dispersibility [11, 12], which then attracted a lot of attention and were widely exploited in sensing, bioimaging, and diagnostic. To improve the selectivity, highly-specific groups or materials were always designed to modify the surface of QDs. Molecularly imprinted polymers (MIPs) contained recognition sites with memory function of shape, size, and functional groups to template for high selectivity, which have attracted excellent attention in solid-phase extraction [13–16]. When MIPs were coated on QDs (QDs@MIPs), a promising sensor is provided with high selectivity and sensitivity and facile operation [17–19]. Among different polymerization methods for MIPs [20–22], sol-gel polymerization, using 3-(aminopropyl) triethoxysilane (APTES) as the functional monomer and tetraethyl orthosilicate (TEOS) as the crosslinker, is the widely selected strategy because of the simple synthesis process and water-compatible capability [22]. The Stöber method and reverse microemulsion method were the two main methods for sol-gel polymerization, and the reverse microemulsion method could control the thickness of MIPs layer better for accessible recognition sites [18, 19, 23]. Up to date, some excellent reports about QDs@MIPs by the reverse microemulsion method have been fabricated. Ensafi et al. prepared QDs@MIPs with average diameter at approximate 40 nm, which were then utilized to sensitively detect thioridazine hydrochloride in plasma within 2 min [18]. Yang et al. fabricated QDs@MIPs with a diameter at about 75 nm and then used them for sensitive and selective determination of tetracycline in fish within 5 min [19]. Wu et al. grafted MIPs onto QDs, and the resultant QDs@MIPs with diameter at about 49 nm could successfully determine malachite green in fish samples within 5 min [23]. Although FA-imprinted polymers have been reported, almost all of them were prepared by copolymerization of allylic functional monomer and crosslinker in the presence of FA under the auxiliary of nitrogen [24–26]. Up to date, QDs@MIPs for sensitive determination of FA have not been reported.

In the current work, a fluorescence sensor based on CdTe-QDs@MIPs was fabricated via the reverse microemulsion method for highly selective and sensitive determination of FA. Preparation and determination conditions were optimized. The resultant CdTe-QDs@MIPs with MIPs layer at about 15 nm were applied to detected FA in fruits based on the charge transfer principle with equilibrium time within 3 min and LOD at 0.85 *μ*g·L^−1^. Therefore, a rapid, selective, and sensitive method for determination of FA was developed.

## 2. Materials and Methods

### 2.1. Reagents and Chemicals

Tellurium powder, thioglycolic acid (TGA), CdCl_2_·2H_2_O, and sodium borohydride (NaBH_4_) were supported by Aladdin Reagent Co., Ltd. (Shanghai, China). TEOS, APTES, NaOH, anhydrous methanol, acetone, Triton X-100, Tris (hydroxymethyl) aminomethane, and anhydrous methanol were acquired from Sinopharm Chemical Reagent Co., Ltd. (Shanghai, China). Standards, ferulic acid (FA), chlorogenic acid (CGA), 4-hydroxybenzoic acid (4-HBA), caffeic acid (CA), *p*-coumaric acid (pCA), vanillic acid (VA), and protocatechuic aldehyde (PA) were ordered from Xiya Reagent Co., Ltd. (Chengdu, China). All stock solutions were prepared with ultrapure water (18.2 MΩ) from a Milli-Q water purification system (Millipore, Bedford, MA, USA).

### 2.2. Apparatus

Fluorescence spectroscopy was investigated on a LS-55 fluorescence spectrometer (PerkinElmer Ltd., Washington, USA) and a Fluo Time 100 fluorescence spectrometer (PicoQuant, Germany). UV-vis absorption spectra were recorded using an UV-2600 spectrometer (Shimadzu, Tokyo, Japan). Functional groups were measured on a Nicolet-Avatar 360 Fourier transform infrared (FT-IR) spectrometer within the wavenumber from 400 to 4,000 cm^−1^. Structure and morphology of CdTe-QDs@MIPs were determined on a Tecnai G2 20S-Twin transmission electron microscope (TEM, FEI, Prague, Czech Republic). The average value of the particle size was determined by a particle size analysis (PSA) (Malvern Instruments Ltd., Malvern, UK).

HPLC analysis was carried out on an Agilent 1260 HPLC system with a UV detector at 322 nm (Agilent Technologies, Santa Clara, CA). A Waters SunFire-C_18_ chromatographic column (250 nm × 4.6 mm i.d., 5 *μ*m, Waters, Milford, MA) was selected for separation at 25°C, and 0.3% acetic acid in ultrapure water/acetonitrile (20/80, v/v) was used as mobile phase for isocratic elution with a flow rate of 0.8 ml/min. Sample (20 *μ*l) was injected directly into HPLC after passing through a 0.22 *μ*m polytetrafluoroethylene syringe filter.

### 2.3. Synthesis of CdTe-QDs@MIPs

Fabrication of CdTe-QDs@MIPs included the preparation of TGA-capped CdTe-QDs and surface MIPs. Firstly, TGA-capped CdTe-QDs were synthesized with some modifications according to previous report [16]. Typically, 30.7 mg of CdCl_2_·2H_2_O and 70.0 *μ*l of TGA were dissolved in 100.0 ml of water, and then, 0.5 mol/l of NaOH solution was added dropwise to adjust pH at 9.0. At the same time, under nitrogen atmosphere, 25.5 mg of tellurium powder and 30.3 mg of NaBH_4_ were mixed in 2.0 ml of water, and the mixture was stirred for about 3 h to obtain NaHTe solution. After that, two above-prepared solutions were mixed and refluxed at 90°C for 13 h to obtain TGA-capped CdTe-QDs solution. Subsequently, CdTe-QDs@MIPs were prepared via the reverse microemulsion method. Triton-X (1.8 ml) and cyclohexane (7.5 ml) were mixed in a three-necked flask and stirred for 30 min. Then, TGA-capped CdTe-QDs solution (1.0 ml) and TEOS (50 *μ*l) were added and continuously stirred for 30 min. At this time, FA (34.0 mg) and APTES (22.8 *μ*l) were separately dissolved in anhydrous methanol, and the resultant solutions were slowly dropped into the three-necked flask. Mixtures were kept stirring for 12 h at room temperature. After that, acetone (40.0 *μ*l) was injected to break the microemulsion and mixtures were centrifuged at 9000 rpm for 1 min. The resultant precipitate was extracted with ethanol to remove FA. As a control, CdTe-QDs@nonimprinted polymers (CdTe-QDs@NIPs) were prepared in the absence of template, FA. CdTe-QDs@MIPs and CdTe-QDs@NIPs were both dried under vacuum and stored at 4°C for further use.

### 2.4. Fluorescence Measurement

For fluorescence quenching analysis of FA towards CdTe-QDs@MIPs and CdTe-QDs@NIPs, 0.3 ml of CdTe-QDs@MIPs or CdTe-QDs@NIPs solution (0.8 mg/ml) was dispersed in 2.7 ml of Tris-HCL buffer (10.0 mM and pH 8.0) and then 30.0 *μ*l of FA standard solution with different concentrations was added. After 3 min stirring process, the fluorescence spectra were recorded. In fluorescence detection, the excitation wavelength was set at 380 nm with an emission range of 550−650 nm, the excitation and emission slit width were both at 10 nm, and the photomultiplier tube voltage was tuned to 900 V.

### 2.5. Determination of FA in Fruit Juices

The real sample applicability of the developed method was to analyze FA in pineapple juice and apple juice (Beijing Huiyuan Beverage and Food Co., Ltd., China) from local supermarket in Changsha. Fruit juices were filtered through a 0.22 *μ*m filter and further used without any sample pretreatment. Briefly, 30.0 *μ*l of fruit juices, 0.3 ml of CdTe-QDs@MIPs solution (0.8 mg/ml), and 2.7 ml of Tris-HCL buffer (10 mM, pH 8.0) were mixed and vortexed for 3 min, and then, the fluorescence intensities were recorded with excitation wavelength at 380 nm.

## 3. Results and Discussion

### 3.1. CdTe-QDs@MIPs Synthesis and Characterization

**Figure 1:**
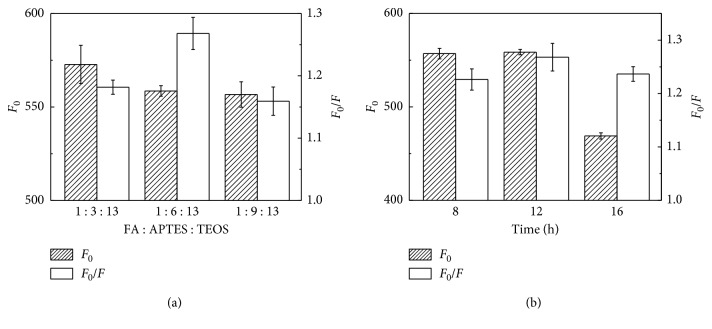
Effect of the ratio of FA : APTES : TEOS (a) and polymerization time (b) on the fluorescence response and quenching efficiency of CdTe-QDs@MIPs (concentration of FA at 10 *μ*g/l).

**Figure 2:**
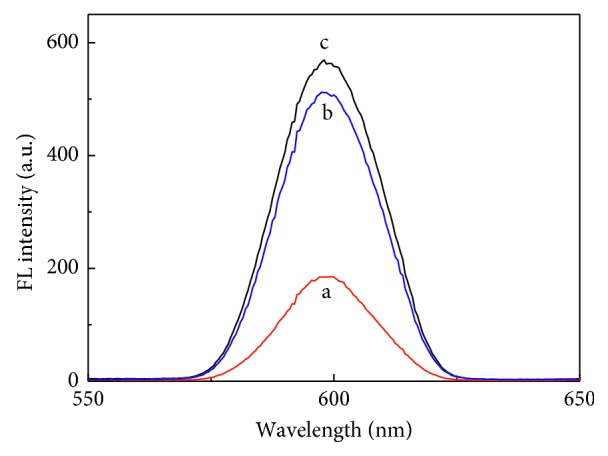
Fluorescence spectra of CdTe-QDs@MIPs before (a) and after (b) removal of FA and fluorescence spectra of CdTe-QDs@NIPs (c).

The reverse microemulsion method was used to prepare CdTe-QDs@MIPs, in which Triton-X and cyclohexane were served as the surfactant and continuous phase, respectively. APTES reacted with FA through hydrogen bonds and electrostatic interaction and hydrolyzed and condensated with TEOS to form FA-imprinted polymers on CdTe-QDs. The fluorescence intensity of CdTe-QDs@MIPs was quenched after binding with FA, which was then recovered after removal of FA. It was obvious that the molar ratio of template-functional monomer-crosslinker was crucial to the recognition ability of MIPs [21, 22]. CdTe-QDs@MIPs with different molar ratios of FA : APTES : TEOS (1 : 3 : 13–1 : 9 : 13) were prepared. Fluorescence intensities of CdTe-QDs@MIPs decreased with the increase of FA content; however, maximum fluorescence quenching efficiency of CdTe-QDs@MIPs was obtained when the molar ratio of FA : APTES : TEOS was 1 : 6 : 13 (Figure 1(a)). Furthermore, polymerization time was optimized (Figure 1(b)). Obviously, fluorescence intensities decreased when polymerization time was over 12 h because of the thicker MIPs shell and more-embedded recognition sites. At the same time, quenching efficiency decreased when the polymerization time was less than 12 min, which was probably because of the less recognition sites in thinner MIPs shell. CdTe-QDs@MIPs with polymerization time at 12 h showed the highest quenching efficiency. Fluorescence intensities of resultant CdTe-QDs@MIPs before and after FA removal were measured. As shown in Figure 2, the maximum emission was about 600 nm when excited at 380 nm and the fluorescence intensities of CdTe-QDs@MIPs after FA removal was 89.7% that of CdTe-QDs@NIPs, which indicated that CdTe-QDs@MIPs were successfully fabricated and could selectively detect FA.

**Figure 3:**
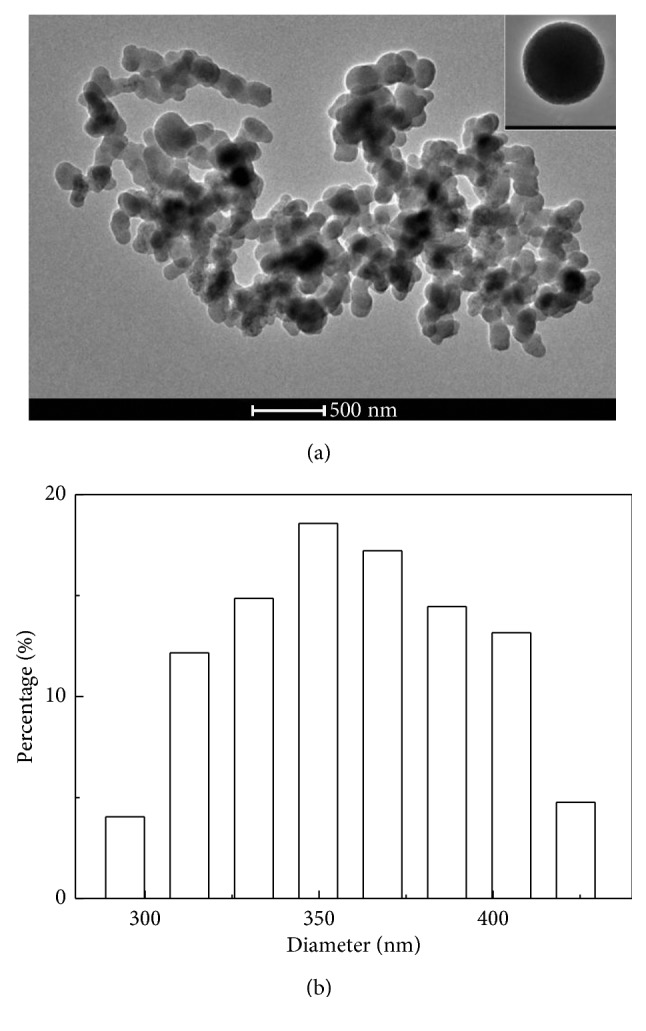
TEM image (a) and PSA histogram (b) of CdTe-QDs@MIPs.

**Figure 4:**
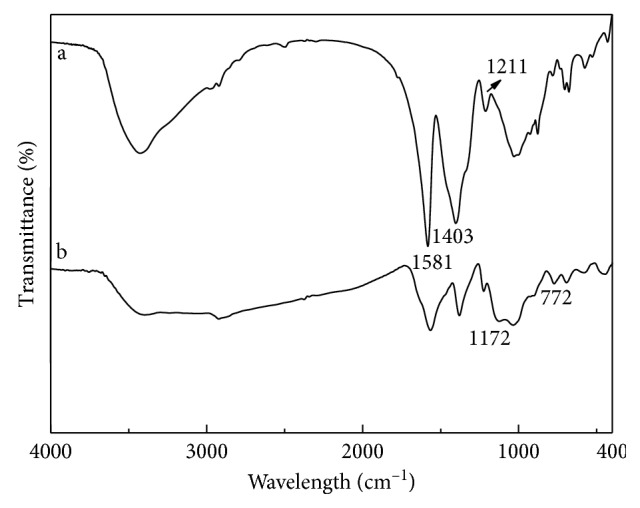
FT-IR spectra of amino-functionalized TGA-capped CdTe-QDs (a) and CdTe-QDs@MIPs (b).

Figure 3 presented the morphology and particle size of CdTe-QDs@MIPs, which indicated that CdTe-QDs@MIPs particles showed a clear core-shell structure with an average diameter of 350 nm and the thickness of MIPs layer at about 15 nm. The reverse microemulsion method can be used in MIPs synthesis with better controlling of particle size than the Stöber method and another sol-gel polymerization method [16, 20–22]. FT-IR spectra were also employed to characterize CdTe-QDs@MIPs (Figure 4). The bands at 1211 cm^−1^ and 1403 cm^−1^ for O−H stretching and C=O stretching and the band at 1581 cm^−1^ for deformation vibration of the carboxylic group ascribed to the modification of TGA on CdTe-QDs (Figure 4(a)). Furthermore, the typical peaks at 1172 cm^−1^ and 772 cm^−1^ were associated with the asymmetric stretching of Si−O−Si and the bending vibration of Si−O (Figure 4(b)), which confirmed the successful preparation of CdTe-QDs@MIPs.

### 3.2. Imprinting Mechanism of CdTe-QDs@MIPs

**Figure 5:**
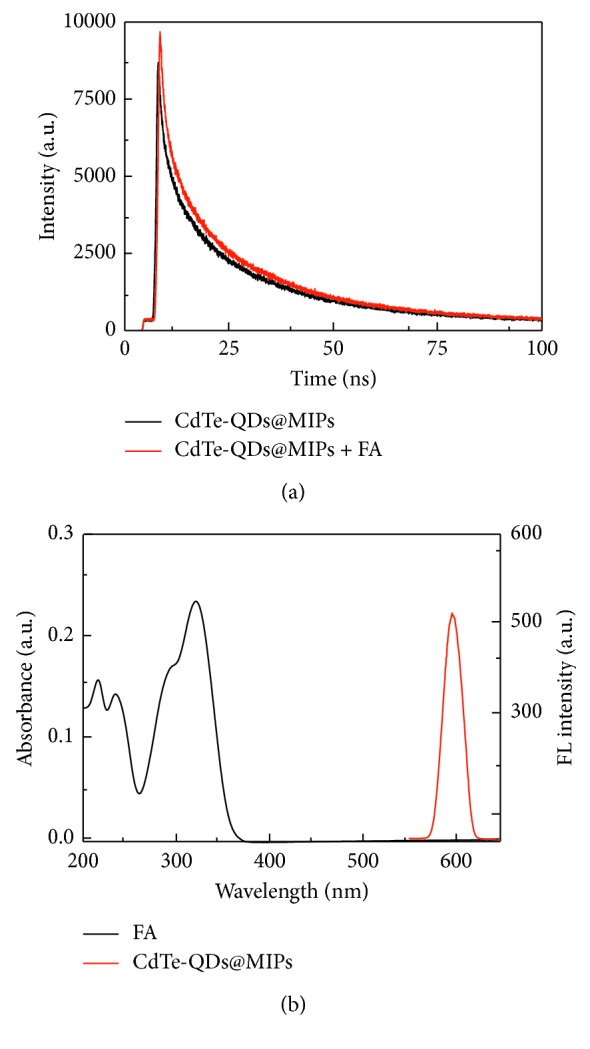
Fluorescence lifetime spectra of CdTe-QDs@MIPs in the absence and presence of FA (a) and UV-vis absorption spectrum of FA and fluorescence emission spectrum of CdTe-QDs@MIPs (b).

As shown in Figure 5(a), the fluorescence lifetimes of CdTe-QDs@MIPs in the absence and presence of FA were 7.98 and 8.35 ns, respectively. The variable fluorescence lifetimes suggested that there existed energy or charge transfer between CdTe-QDs and FA [27].  Figure 5(b) revealed that FA had adsorption bands at 300 and 322 nm, which were close to the band gap of CdTe-QDs [17], and far away from the emission spectrum (600 nm) of CdTe-QDs@MIPs. Therefore, charges in the conduction band of CdTe-QDs could transfer to the lowest unoccupied molecular orbital of FA, and energy resonance transfer was not the possible quenching mechanism. Furthermore, hydroxyl and carboxyl groups in FA could interact with the amino group in APTES through electrostatic interaction and hydrogen bond; therefore, it could be concluded that the observed fluorescence quenching was attributed to the charge transfer between FA and CdTe-QDs.

### 3.3. Optimization of Determination Conditions for FA

During fluorescence detection, several factors, such as the CdTe-QDs@MIPs concentration, pH, and adsorption time, can affect the linear rang and sensitivity, and then, they were optimized.

**Figure 6:**
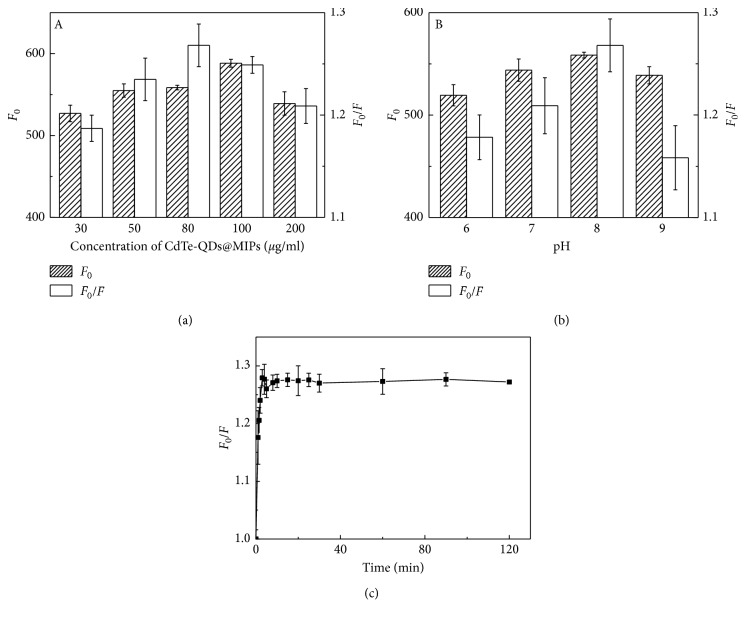
Effect of the concentration of CdTe-QDs@MIPs (a), pH (b), and incubation time (c) on the response of CdTe-QDs@MIPs to FA (concentration of FA at 10 *μ*g/l).

The CdTe-QDs@MIPs concentration was very important for fluorescence intensity and quenching efficiency. It has been reported that a narrow linear range came from too low CdTe-QDs@MIPs concentrations and low sensitivity happened by using too high CdTe-QDs@MIPs concentrations [18, 19]. As shown in Figure 6(a), the fluorescence intensities increased when the CdTe-QDs@MIPs increased from 30 to 100 *μ*g/ml and decreased from 100 to 200 *μ*g/ml. In higher concentrations, slight agglomeration of CdTe-QDs@MIPs would happen. Highest quenching efficiency was achieved with a CdTe-QDs@MIPs concentration at 80 *μ*g/ml.

The solution pH values presented a great effect on the fluorescence intensities of CdTe-QDs@MIPs and the recognition of FA (Figure 6(b)). Among noncovalent interactions, binding energy of electrostatic interaction is higher than that of hydrogen bonding and *π*-*π* stacking. Therefore, by electrostatic interaction, template-functional monomer complex is more stable and recognition ability is stronger [28]. Here, surface MIPs were synthesized at pH 8.0 because of the electrostatic interaction and hydrogen bonding between FA and APTES. However, imprinting silica layer was ionized at higher alkaline conditions, which resulted in the poor recognition for FA. Therefore, fluorescence intensity and quenching efficiency of CdTe-QDs@MIPs decreased at pH ≥ 8. Finally, Tris buffer (10 mM, pH 8.0) was chosen as the optimum pH value for high fluorescence intensity and quenching efficiency.

The influence of incubation time of CdTe-QDs@MIPs with FA was important for the complete binding. As presented in Figure 6(c), quenching efficiency of CdTe-QDs@MIPs increased sharply from 0 to 3 min and after that tended to be stable. Therefore, incubation time was set at 3 min. The fast mass transfer rate was assigned to surface thin MIPs layers.

### 3.4. Specificity Study

**Figure 7:**
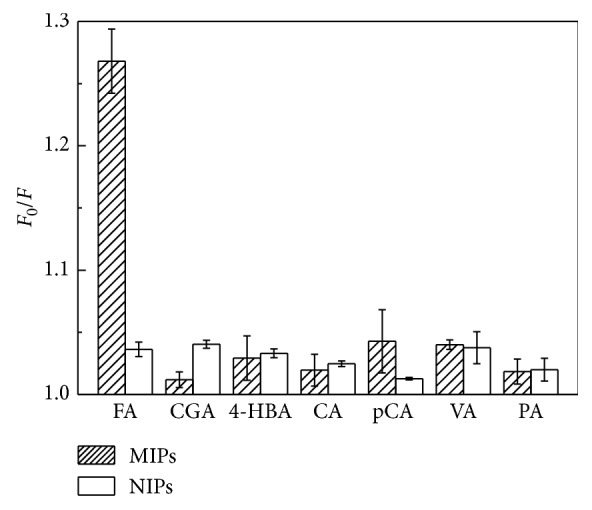
Quenching efficiencies of FA, CGA, 4-HBA, CA, pCA, VA, and PA (each concentration at 10 *μ*g/l) on CdTe-QDs@MIPs and CdTe-QDs@NIPs.

Selectivity was one of the most important issues in fabrication of optical sensors. It was reported that phenylpropanoid acids (e.g., CA, pCA, and FA) could simultaneously quench the fluorescence of CdTe-QDs [11, 29]. Bare CdTe-QDs could not selectively detect phenylpropanoid acid; therefore, surface modification should be fabricated. Six structural analogues, chlorogenic acid (CGA), 4-hydroxybenzoic acid (4-HBA), caffeic acid (CA), *p*-coumaric acid (pCA), vanillic acid (VA), and protocatechuic aldehyde (PA) with a concentration at 10 *μ*g/l were selected and investigated to estimate the selectivity of CdTe-QDs@MIPs and CdTe-QDs@NIPs. As illustrated in Figure 7, the quenching efficiency of FA was obviously different between CdTe-QDs@MIPs (*F*_0_/*F*, 1.27) and CdTe-QDs@NIPs (*F*_0_/*F*, 1.04), which verified that CdTe-QDs@MIPs had specific recognition ability to FA. However, CGA, 4-HBA, CA, pCA, VA, and PA quenching efficiencies all close to 1.00 were not easy to identify. Results further demonstrated that the recognition ability of CdTe-QDs@MIPs was based on the recognition sites.

### 3.5. Method Validation

**Figure 8:**
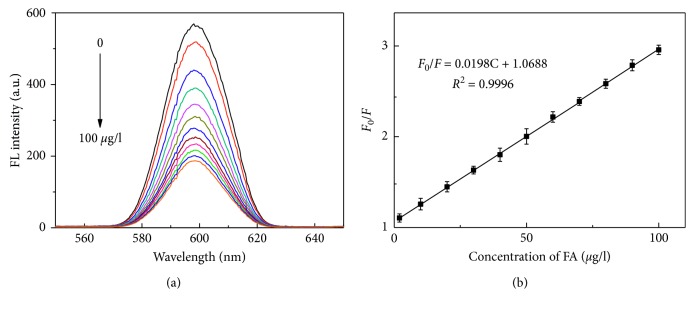
Fluorescence spectra of CdTe-QDs@MIPs in the presence of different concentrations of FA (2–100 *μ*g/l) (a) and the Stern–Volmer curve (b).

Linearity, limit of detection (LOD), limit of quantification (LOQ), precision, and specificity were determined to investigate the method validation. The fluorescence intensities of CdTe-QDs@MIPs decreased with the increase of FA concentrations (Figure 8(a)). The fluorescence quenching efficiencies followed Stern–Volmer equation: *F*_0_/*F* = 1 + *K*_sv_[*C*], where *K*_sv_ and [*C*] were the quenching constant and FA concentrations. Furthermore, a good linearity (*F*_0_/*F* = 1.0688 + 0.0198 C) was observed at the concentration range from 2 to 100 *μ*g/l with coefficient (*R*^2^) at 0.9996 (Figure 8(b)). LOD (0.85 *μ*g/l) and LOQ (2.81 *μ*g/l) were achieved for FA. Intraday RSD and interday RSD values over a five-day span were 0.62% and 1.61%, respectively, indicating the high reproducibility. Accordingly, the developed method was sufficiently practical for selective quantification of FA. To further investigate the developed performance, the results with other methods were compared [5–7, 9–11, 24]. As shown in Table 1, different methods have different superiorities and disadvantages. It can be seen the developed method was one of the most sensitive for FA quantification.

###  3.6. Real Sample Measurement

The practicability of the developed CdTe-QDs@MIPs-based sensor was further evaluated for quantitative analysis of FA in pineapple juice and apple juice. In addition to dilution, no sample pretreatment procedures were required. Recoveries of FA ranged from 91.8% to 110.3% with excellent RSD values less than 6.6% (Table 2). Finally, FA was estimated in pineapple juice at a concentration of 724.6 ± 29.5 *μ*g/l, and no FA was detected in apple juice.

In the Pharmacopoeia of the People's Republic of China, FA was always quantified by HPLC-UV. Quantitative analysis of FA in pineapple juice and apple juice has been developed in our group by HPLC after MIPs extraction [15]. Results indicated the concentrations of FA in pineapple juice and apple juice were 683.3 ± 33.1 and 0 *μ*g/l, which were consistent with those determined by our developed CdTe-QDs@MIPs-based method.

## 4. Conclusions

In summary, a fluorescent probe based on CdTe-QDs@MIPs was successfully synthesized. The molecular imprinting technique validated the selectivity of CdTe-QDs@MIPs. The thin MIPs layer made the fluorescent detection fast (response time for 3 min) and sensitivity high (LOD for 0.85 *μ*g/l). With the merits, the practicability of the proposed fluorescent method was verified by detection of FA in juices. The proposed strategy may offer an optimum approach to develop rapid and sensitive methods for biological, environmental, and clinical diagnostics applications.

## Figures and Tables

**Table 1:** Comparison of the developed method with other reported methods for FA determination.

Detection scheme	Linear range (*μ*g/l)	LOD (*μ*g/l)	Reference
HPLC-DAD	781.25–2.5 × 10^4^	39.0	[5]
HPLC-MS	10–4,000	10.0	[6]
Electrochemical	15.92–4.27 × 10^4^	1.94	[7]
Raman spectroscopy	0.49–1455	0.19	[9]
Chemiluminescence	300–1.0 × 10^5^	80.0	[10]
CdTe-QDs	35.15–2.82 × 10^4^	9.84	[11]
MIPs‒HPLC	100–1.0 × 10^5^	45.0	[24]
CdTe-QDs@MIPs	2.0–100.0	0.85	This work

**Table 2:** Recoveries of FA in fruit juices by CdTe-QDs@MIPs-based sensor (*n*=3).

Sample	Added (*μ*g/l)	Found^a^ (*μ*g/l)	Recovery (%)	RSD (%)
Pineapple juice	5.00	5.15	103.0	5.7
	20.00	18.93	94.6	6.6
	50.00	46.79	93.6	3.2

Apple juice	5.00	5.48	110.3	2.9
	20.00	19.20	96.0	5.2
	50.00	45.90	91.8	3.1

^a^Mean of three determinations.

## Data Availability

The data used to support the findings of this study are available from the corresponding author upon request.
